# SesamumGDB: a comprehensive platform for *Sesamum* genetics and genomics analysis

**DOI:** 10.1093/database/baae105

**Published:** 2024-10-19

**Authors:** Cao Hengchun, Guo Hui, Yang Weifei, Li Guiting, Ju Ming, Duan Yinghui, Tian Qiuzhen, Ma Qin, Feng Xiaoxu, Zhang Zhanyou, Zhang Haiyang, Miao Hongmei

**Affiliations:** The Shennong Laboratory, 116 Huayuan Road, Zhengzhou, Henan 450002, China; Henan Sesame Research Center, Henan Academy of Agricultural Sciences, 116 Huayuan Road, Zhengzhou, Henan 450002, China; Key Laboratory of Specific Oilseed Crops Genomics of Henan Province, Henan Sesame Research Center, Henan Academy of Agricultural Sciences, 116 Huayuan Road, Zhengzhou, Henan 450002, China; Henan Sesame Research Center, Henan Academy of Agricultural Sciences, 116 Huayuan Road, Zhengzhou, Henan 450002, China; Key Laboratory of Specific Oilseed Crops Genomics of Henan Province, Henan Sesame Research Center, Henan Academy of Agricultural Sciences, 116 Huayuan Road, Zhengzhou, Henan 450002, China; The Shennong Laboratory, 116 Huayuan Road, Zhengzhou, Henan 450002, China; Henan Sesame Research Center, Henan Academy of Agricultural Sciences, 116 Huayuan Road, Zhengzhou, Henan 450002, China; Key Laboratory of Specific Oilseed Crops Genomics of Henan Province, Henan Sesame Research Center, Henan Academy of Agricultural Sciences, 116 Huayuan Road, Zhengzhou, Henan 450002, China; The Shennong Laboratory, 116 Huayuan Road, Zhengzhou, Henan 450002, China; Henan Sesame Research Center, Henan Academy of Agricultural Sciences, 116 Huayuan Road, Zhengzhou, Henan 450002, China; Key Laboratory of Specific Oilseed Crops Genomics of Henan Province, Henan Sesame Research Center, Henan Academy of Agricultural Sciences, 116 Huayuan Road, Zhengzhou, Henan 450002, China; The Shennong Laboratory, 116 Huayuan Road, Zhengzhou, Henan 450002, China; Henan Sesame Research Center, Henan Academy of Agricultural Sciences, 116 Huayuan Road, Zhengzhou, Henan 450002, China; Key Laboratory of Specific Oilseed Crops Genomics of Henan Province, Henan Sesame Research Center, Henan Academy of Agricultural Sciences, 116 Huayuan Road, Zhengzhou, Henan 450002, China; The Shennong Laboratory, 116 Huayuan Road, Zhengzhou, Henan 450002, China; Henan Sesame Research Center, Henan Academy of Agricultural Sciences, 116 Huayuan Road, Zhengzhou, Henan 450002, China; Key Laboratory of Specific Oilseed Crops Genomics of Henan Province, Henan Sesame Research Center, Henan Academy of Agricultural Sciences, 116 Huayuan Road, Zhengzhou, Henan 450002, China; The Shennong Laboratory, 116 Huayuan Road, Zhengzhou, Henan 450002, China; Henan Sesame Research Center, Henan Academy of Agricultural Sciences, 116 Huayuan Road, Zhengzhou, Henan 450002, China; Key Laboratory of Specific Oilseed Crops Genomics of Henan Province, Henan Sesame Research Center, Henan Academy of Agricultural Sciences, 116 Huayuan Road, Zhengzhou, Henan 450002, China; The Shennong Laboratory, 116 Huayuan Road, Zhengzhou, Henan 450002, China; Henan Sesame Research Center, Henan Academy of Agricultural Sciences, 116 Huayuan Road, Zhengzhou, Henan 450002, China; Key Laboratory of Specific Oilseed Crops Genomics of Henan Province, Henan Sesame Research Center, Henan Academy of Agricultural Sciences, 116 Huayuan Road, Zhengzhou, Henan 450002, China; The Shennong Laboratory, 116 Huayuan Road, Zhengzhou, Henan 450002, China; Henan Sesame Research Center, Henan Academy of Agricultural Sciences, 116 Huayuan Road, Zhengzhou, Henan 450002, China; Key Laboratory of Specific Oilseed Crops Genomics of Henan Province, Henan Sesame Research Center, Henan Academy of Agricultural Sciences, 116 Huayuan Road, Zhengzhou, Henan 450002, China; The Shennong Laboratory, 116 Huayuan Road, Zhengzhou, Henan 450002, China; Henan Sesame Research Center, Henan Academy of Agricultural Sciences, 116 Huayuan Road, Zhengzhou, Henan 450002, China; Key Laboratory of Specific Oilseed Crops Genomics of Henan Province, Henan Sesame Research Center, Henan Academy of Agricultural Sciences, 116 Huayuan Road, Zhengzhou, Henan 450002, China; The Shennong Laboratory, 116 Huayuan Road, Zhengzhou, Henan 450002, China; Henan Sesame Research Center, Henan Academy of Agricultural Sciences, 116 Huayuan Road, Zhengzhou, Henan 450002, China; Key Laboratory of Specific Oilseed Crops Genomics of Henan Province, Henan Sesame Research Center, Henan Academy of Agricultural Sciences, 116 Huayuan Road, Zhengzhou, Henan 450002, China; The Shennong Laboratory, 116 Huayuan Road, Zhengzhou, Henan 450002, China; Henan Sesame Research Center, Henan Academy of Agricultural Sciences, 116 Huayuan Road, Zhengzhou, Henan 450002, China; Key Laboratory of Specific Oilseed Crops Genomics of Henan Province, Henan Sesame Research Center, Henan Academy of Agricultural Sciences, 116 Huayuan Road, Zhengzhou, Henan 450002, China

## Abstract

Sesame (*Sesamum indicum* L., 2*n* = 26) is a crucial oilseed crop cultivated worldwide. The ancient evolutionary position of the *Sesamum* genus highlights its value for genomics and molecular genetics research among the angiosperms of other genera. However, *Sesamum* is considered a small orphan genus with only a few genomic databases for cultivated sesame to date. The urgent need to construct comprehensive, curated genome databases that include genus-specific gene resources for both cultivated and wild *Sesamum* species is being recognized. In response, we developed Sesamum Genomics Database (SesamumGDB), a user-friendly genomic database that integrates extensive genomic resources from two cultivated sesame varieties (*S. indicum*) and seven wild *Sesamum* species, covering all three chromosome groups (2*n* = 26, 32, and 64). This database showcases a total of 352 471 genes, including 6026 related to lipid metabolism and 17 625 transcription factors within *Sesamum*. Equipped with an array of bioinformatics tools such as BLAST (basic local alignment search tool) and JBrowse (the Javascript browser), SesamumGDB facilitates data downloading, screening, visualization, and analysis. As the first centralized *Sesamum* genome database, SesamumGDB offers extensive insights into the genomics and genetics of sesame, potentially enhancing the molecular breeding of sesame and other oilseed crops in the future.

**Database URL**: http://www.sgbdb.com/sgdb/

## Introduction

Sesame (*Sesamum indicum* L., 2*n* = 26) is an ancient and crucial oilseed crop, notably known for its high oil content and quality, and is prominent worldwide [1, 2]. Sesame, which originated in Africa and is now widely cultivated in the tropical and subtropical regions of Asia, Africa, and South America, has a history of cultivation dating back to ∼3000–50 bc in the Harappa Valley of the Indian subcontinent [3]. The genus *Sesamum*, classified within the Pedaliaceae family, is notable for its diversity, comprising over 30 wild species in addition to the single cultivated species *S. indicum* L [4, 5]. Compared with the cultivated sesame, some wild species, such as *Sesamum calycinum*, *Sesamum latifolium*, and *Sesamum radiatum*, exhibit higher resistance and tolerance to biotic and abiotic stresses, making them valuable genetic resources for advancing modern molecular breeding in sesame [6].

With the rapid advancement of sequencing technologies, substantial progresses in sesame genomics and molecular breeding have been achieved over the past decade. Notably, the completion of the Sesame Genome Project (SGP) in 2020 provided essential genome data for *Sesamum* genomics research [7, 8]. Pan-genome analysis of chromosome-scaled genomes from the cultivated sesame and six wild *Sesamum* species provided clear evidence of genome evolution and domestication. For the *Sesamum* genus, four chromosome groups (i.e. groups A, B, C, and D) were named and differentiated for the first time. Both whole-genome duplication and whole-genome triplication events were identified in the assembled seven *Sesamum* species. A typical allotetraploidization event occurred in *S. radiatum* (2*n* = 4*x* = 64), significantly contributing to the divergence of species within the *Sesamum* genus [3]. Genome evolution analysis revealed losses and expansions in numerous gene families that regulate key agronomic traits and crucial processes, such as plant type, inflorescence meristem development, fatty acid biosynthesis and metabolism, and resistance to *Fusarium* wilt disease in sesame [3]. To date, multiple *de novo* assembled genome maps of cultivated sesame (four varieties) and seven wild species have been published [9–12].

To enhance the accessibility of sesame genome data, various raw sequencing data, and genomics information, several sesame genome databases have been established. These databases can be categorized into three groups on the basis of their primary resources. The first category includes online genome databases, such as Sinbase and the SGP database (http://sesamum.org/), which focus on *de novo* assembled genome data and were constructed in 2015 and 2013, respectively [13]. The second category, reported between 2015 and 2021, comprises databases dedicated to sesame functional genomics, including SesameFG, SiGeDiD, and SesameHapMap [14–16]. The third category includes molecular marker databases, such as GinMicrosatDb and SisatBase, which were also published during this period [17, 18]. However, all these genome databases cover only the cultivated sesame, and most are not continuously accessible by users worldwide.

In this study, we integrated the latest genomic resources from the SGP with additional public datasets, which include two cultivated sesame varieties (Yuzhi11 and Zhongzhi13) and seven wild *Sesamum* species. Our objective was to develop a novel and user-friendly genome database for *Sesamum* genus, named Sesamum Genomics Database (SesamumGDB). This database includes a suite of practical bioinformatics online tools and provides a comprehensive genomic resource that focuses on specific gene families, including lipid biosynthesis and metabolism, and transcription factors (TFs). We identified and systematically cataloged 6026 genes related to lipid metabolism and 17 625 TF genes across 58 types in 8 *Sesamum* species. This comprehensive platform is poised to make significant contributions to sesame genomics research and enhance molecular breeding efforts in the future.

## Materials and methods

### Data sources

The database encompasses nine high-quality *de novo* genomic datasets, representing two cultivated sesame varieties, Yuzhi 11 and Zhongzhi 13 (*S. indicum*, 2*n* = 2*x* = 26), and seven wild species, namely *S. alatum, S. angustifolium, S. latifolium, S. calycinum*, and *S. angolense* (all 2*n* = 2*x* = 32), along with the tetraploid species *S. radiatum* and *S. schinzianum* (both 2*n* = 4*x* = 64). A comprehensive overview is provided in Table 1, which details all genomic data that are made publicly accessible.

**Table 1.** Catalog of genome information of two cultivated sesame varieties and seven wild relatives in SesamumGDB

Species	Variety name	Type	Karyotype	Genome size (Mb)	N50 (Mb)	Gene number	NCBI accession	Reference
*S. indicum*	Yuzhi11	Cultivated	2*n* = 26	346.8	22.9	31 462	GCA_003268515.1	[6]
*S. indicum*	Zhongzhi13	Cultivated	2*n* = 26	270.3	17.3	35 410	GCF_000512975.1	[7]
*S. alatum*	3651	Wild	2*n* = 26	528.0	40.5	25 722	GCA_034509735.1	[3]
*S. angustifolium*	G01	Wild	2*n* = 32	300.7	11.6	32 646	JACGWK000000000	
*S. latifolium*	Ken1	Wild	2*n* = 32	369.0	22.2	42 114	JACGWN000000000	
*S. calycinum*	Ken8	Wild	2*n* = 32	313.0	14.3	31 417	JACGWM000000000	
*S. angolense*	K16	Wild	2*n* = 32	300.8	24.1	31 091	GCA_034509725.1	
*S. radiatum*	G02	Wild	2*n* = 64	668.4	19.5	68 397	JACGWJ000000000	
*S. schinzianum*	Gangguo	Wild	2*n* = 64	704.5	19.7	54 212	GCA_027475655.1	[10]

### Genome reannotation

To ensure the consistency and reliability of gene annotation and prediction across the nine *Sesamum* genomes, we conducted a comprehensive reannotation. Tandem repeats in each genome were identified using Tandem Repeats Finder (v4.09) with default settings [19]. Transposable elements were detected using LTR_finder (v1.07), LTR_retriever (V2.9.0), and RepeatModeler (v2.0.3) [20–22]. Whole-genome repeat sequences were then masked using RepeatMasker (v4.1.1) [23, 24]. Gene structure prediction was combined with *ab initio*-, homology-, and RNA-seq-based methods. Braker2 and tblastn were employed for *ab initio*- and homology-based gene predictions, respectively [25].

We predicted homologous proteins between the assembled *Sesamum* genomes and four model plants closely related to sesame, including *Arabidopsis thaliana, Solanum lycopersicum, Vitis vinifera L*., and *Mimulus guttatus* (Supplementary Table S1). This was performed using tblastn, with an *e*-value of 1e-5. Hisat2 (v2.1.0), Stringtie (v1.3.4), and PASA (v2.3.3) were used for RNA-seq-based gene prediction [26, 27]. Furthermore, EVidenceModeler was used to integrate the results of gene predictions and obtain a consensus gene set [28]. Throughout the gene prediction process, the Non-Redundant Protein Sequence Database, Kyoto Encyclopedia of Genes and Genomes (KEGG), SwissProt, and Gene Ontology (GO) databases were comprehensively used for gene function annotation [29–32]. Noncoding RNAs, including rRNAs, small RNA, *cis*-regulatory elements, and tRNA, were identified using a combination of Infernal (v1.1.2), tRNAscan-SE (v2.0.10), and RNAmmer programs and the Rfam database [33, 34].

### Gene family identification

To accurately identify and functionally annotate gene families within the *Sesamum* genomes, we employed two methodologies: emapper and pfam_scan. The primary protein sequences from the eight species were annotated using emapper (v2.1.7) and pfam_scan (v1.6), both set to their default parameters [35, 36].

### TF identification

The web tools in the Plant Transcription Factor Database (PlantTFDB) were used to identify TFs in the nine genomes [37]. We conducted the TF predictions using the protein sequences derived from these genomes on the Transcription Factor Prediction page, adhering to the default settings to maintain consistency. After completing the prediction process, we systematically organized and enumerated the identified TFs according to their nomenclature, facilitating a clear and structured classification of the results.

### Identification of genes related to lipid metabolism

The online tool Mercator4 (v6.0) was used to identify genes related to lipid biosynthesis and metabolism in the *Sesamum* genomes [38]. We uploaded the protein sequences through the FASTA (Fast All Sequences in A) validation process and utilized the default settings on the protein annotation interface to ensure a standardized analysis. Once the analysis was complete, we engaged in a thorough review of the results, selectively identifying genes that fell under the “Lipid metabolism” category. Subsequently, these genes were methodically categorized based on their involvement in the specific subprocesses of lipid metabolism, allowing for a systematic and detailed classification of the identified genes related to lipid pathways.

### Integration of bioinformatics tools with the SesamumGDB

We integrated the SequenceServer with the SesamumGDB to deliver a robust BLAST (basic local alignment search tool) service and provide a user-friendly experience. As an advanced front-end, the SesamumGDB could be used to perform sequence alignment and search [39]. Furthermore, the latest version of JBrowse (the Javascript browser) 2 was used for constructing SesamumGDB and comprehensively visualizing all accessible genomes [40].

### SesamumGDB implementation

The SesamumGDB was developed using the classic LAMP (Linux + Apache + MySQL (My Structured Query Language)+ PHP) stack within a RedHat system on Centos 7.3 environment. This database provides user-friendly web pages for data searching and browsing. The web interface is built with HTML5 and PHP (v5.4) and runs on an Apache web server. The backend is supported by MariaDB (v5.5.68), which houses several interrelated relational databases, including those for lipid metabolism-related genes. The database interfacing and the common gateway interface are programmed in Perl. SesamumGDB is hosted on a World Wide Web server, offering internet access via a web client. The browsers recommended for accessing the database are Google Chrome and Internet Explorer 10.0+ (or higher).

## Results

**Figure 1.**
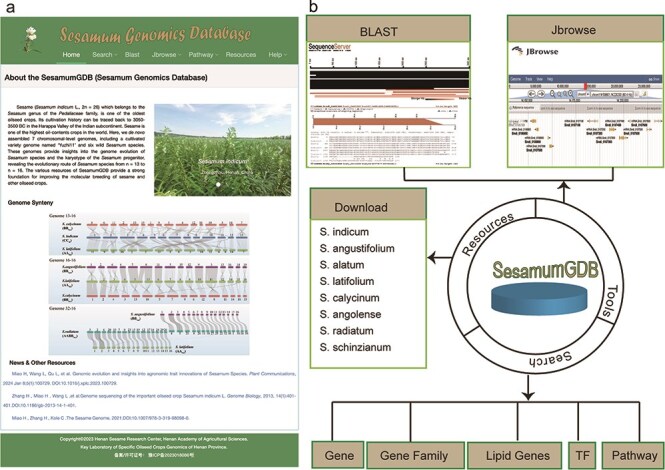
Overview of SesamumGDB, inculding the homepage screenshot (a) and the foundational functions and useful tools (b).

Utilizing a diverse dataset encompassing nine genomes and employing an advanced search platform, we developed SesamumGDB, a pioneering genome database dedicated to the *Sesamum* genus (Fig. 1a). SesamumGDB provides extensive genomic information on individual genes, gene families, TFs, and genes related to lipid biosynthesis and metabolism. Moreover, this database features a robust search catalog that facilitates data downloads, searches, and browsing, including homolog BLAST, GO term, and KEGG pathway screening. In addition, the platform supports dataset visualization and navigation, presenting all data and search results in a user-friendly interface (Fig. 1b). The detailed architecture and functionalities of SesamumGDB are presented in the subsequent sections.

### Details of SesamumGDB

SesamumGDB includes 352 471 genes derived from 9 genomes, involving those of 2 cultivated sesame varieties (Yuzhi 11 and Zhongzhi 13) and 7 wild *Sesamum* species (Table 1). To ensure the reliability of genome information, all nine genomes were reannotated, incorporating data from the GO and KEGG databases, providing a richer context for gene functions and pathways. The database contained a vast array of 317 385 gene families (Supplementary Table S4). Given that sesame is a crucial oilseed crop, special attention was paid to gene families related to energy storage and regulation. Using the Mecrator software for protein annotation, 6026 genes associated with lipid biosynthesis and metabolism were identified (Supplementary Table S3). This comprehensive categorization serves as a valuable resource for further research, which can facilitate the exploration of evolutionary connections and functional commonalities among these gene families in *Sesamum*.

**Figure 2.**
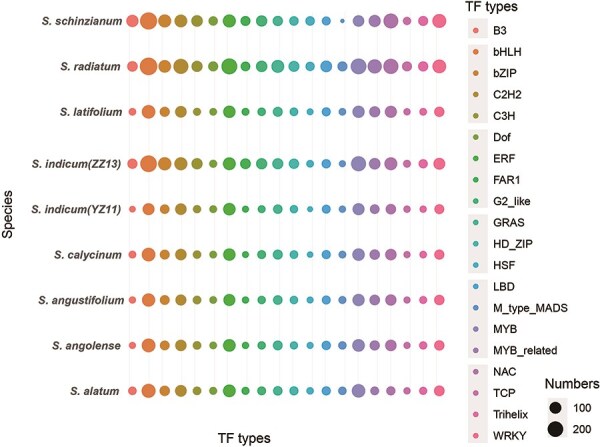
Comparative distribution of the top 20 TFs in SesamumGDB.

The data underwent detailed refinement, enabling systematic screening and cataloging of TF genes across the nine genomes (Fig. 2, Supplementary Table S2). A total of 17 625 TF entries were classified into 58 categories. This curated collection highlights the key regulatory components that play a crucial role in the complex regulation of gene expression in *Sesamum*.

### Searching and browsing genes and genomic features

**Figure 3.**
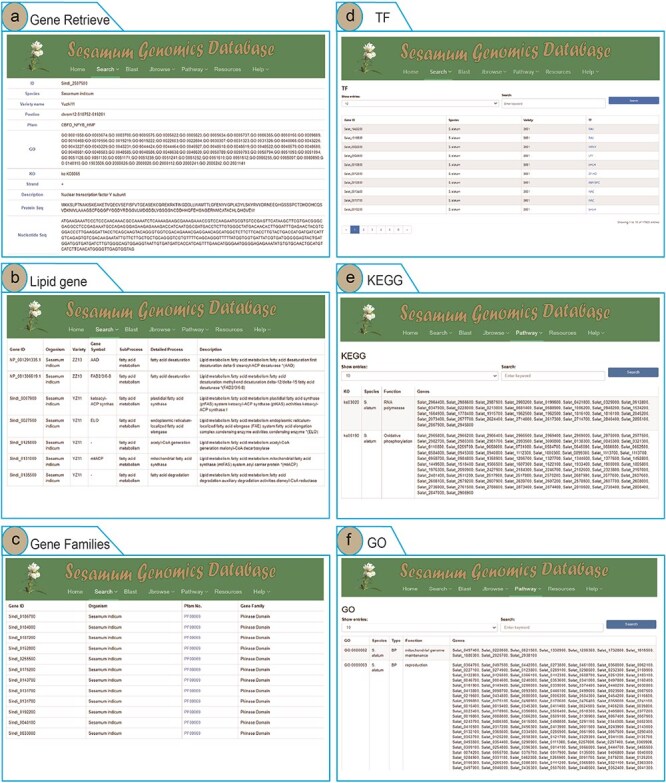
Genomic feature and exploration examples of SesamumGDB, including gene retrieve by species name and gene ID (a), targeted search for lipid metabolism genes (b), gene family discovery by species and Pfam classification (c), TFs in *Sesamum* genomes (d), KEGG pathways browsing (e), and GO terms annotations survey (f).

To enhance user access to *Sesamum* genome data and highlight the advantages of the SesamumGDB database, we developed four search terms and integrated them into the “Search” menu. Users can retrieve information on *Sesamum*-specific genes, lipid-related genes, TF genes, and gene families by using the Gene Retrieve module (Fig. 3). In the “Gene Retrieve” module, users can explore genes by species classification or by entering specific gene IDs. The primary output page provides an exhaustive overview of each gene, including details such as gene ID, species, variety, genomic location, strand, and comprehensive annotations from Pfam, GO, and KEGG databases. Moreover, the database displays the description of genes and their corresponding nucleotide and protein sequences (Fig. 3a).

The search functions in the “Lipid Genes” and “Gene Families” modules are similar, allowing users to select a species and enter specific subtypes of lipid metabolism pathways or gene families into the provided text field. Upon submitting a query, the system generates a detailed report of the search results (Fig. 3b and c).

In the “Transcription Factor” module, users can access all TFs by searching via gene IDs, species, variety, or TF names (Fig. 3d). The interface also allows users to customize the number of entries displayed per page. Additionally, by clicking on the TF names, users can directly hyperlink to the PlantTFDB website, where they can retrieve detailed information regarding the specific TF protein [37].

### Homolog blast

**Figure 4.**
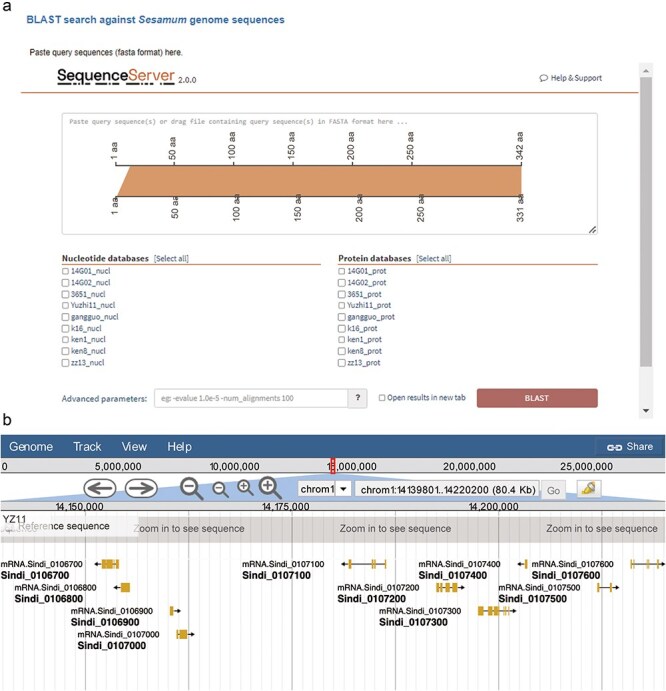
Integrated bioinformatic platforms in SesamumGDB, featuring SequenceServer for gene-specific searches (a) and JBrowse for detailed *Sesamum* genome annotation review (b).

We integrated the SequenceServer software (version 2.0.0) into SesamumGDB to provide a user-friendly interface for conducting homolog BLAST analyses and visualizing the results (Fig. 4a). Users can either paste their query sequence(s) directly or drag and drop a file containing the sequence(s) in the FASTA format into the designated input field. They can then select from a range selection of databases specifically curated for homology search, each constructed using the coding DNA sequences (CDS) and protein sequences from the nine genomes housed within SesamumGDB. The BLAST analysis was streamlined with other programs (BLASTN, BLASTP, BLASTX, tBLASTN, and tBLASTX) and automatically configured to match the nature of the submitted query sequence and the selected database. The platform offers default parameter settings; however, users can customize these settings through an “Advanced parameters” box, allowing them to adjust the *e*-value threshold, scoring matrix, and output formatting to meet their specific research needs. The alignment results are then elegantly presented using various visualization techniques. These visual representations, which are informative and available for downloaded in both scalable vector graphics and Portable Network Graphics (PNG) formats, are of high-quality and can be used directly for presentations and further analysis.

### JBrowse

Apart from searching by gene IDs or genomic locations, SesamumGDB enables the advanced exploration of nine high-quality *Sesamum* genomes through the integration of JBrowse 2. Due to its ability to rapidly visualize and navigate large-scale genomic datasets, JBrowse 2 enhances user interaction with each genome featured in the SesamumGDB (Fig. 4b). Various implemented tracks facilitate the browsing of key genomic features, including genome sequences, CDSs, protein-coding gene models, and exon regions. Users can conveniently isolate a specific chromosome for in-depth examination of the genomic features. The interactive nature of JBrowse 2 facilitates the straightforward selection of elements within any given track. Once an element is selected, an information panel instantly appears to the right of the genome browser interface, presenting the essential details such as gene name, position, length, and sequences. This user-centric design ensures a streamlined experience catering to the diverse needs of the research community.

### GO terms and KEGG pathways

In the SesamumGDB, the “Pathway” menu includes GO and KEGG modules, both of which are crucial for elucidating the functions of protein-coding genes. All genes across the nine genomes were annotated using the eggNOG database (v 5.0.2), allowing for the construction of a comprehensive set of GO terms and KEGG pathways for *Sesamum* genes [41]. The database presents a total of 100 719 GO terms for 153 690 genes and 1296 KEGG pathways for 76 171 genes (Supplementary Table S5).

The “KEGG” module in SesamumGDB offers a straightforward search mechanism, where users can input keywords such as gene ID, species name, KEGG Orthology (KO) number, or functions derived from biological experiments and bioinformatics analysis. These inputs facilitate the rapid retrieval of KEGG-related data. All results are displayed in a tabular format (Fig. 3e). Additionally, by clicking on a KO number, users can access a direct hyperlink to the KEGG Pathway Database (https://www.kegg.jp/kegg/pathway.html) [31], where a detailed pathway map for the specified KO number is presented.

The “GO’”module in SesamumGDB parallels the functionality of the “KEGG” module, allowing users to retrieve GO terms and directly access more detailed information via hyperlinks to the AmiGO2 database for a specific GO term [42]. This streamlined approach ensures easy accessibility to gene function annotation information and the broader biological context covered by these databases (Fig. 3f).

### Data download

To ensure comprehensive access to the genomic resources, SesamumGDB includes a “Resource” page where all processed data related to the nine sesame genomes are readily available for download. These data include a wide range of genomic sequences, CDS, complementary DNA, protein sequences, and detailed annotations. To enhance the downloading experience and to ensure swift data retrieval, all datasets were systematically organized and stored in the compressed zip file format. This approach not only ensures a fast download but also allows researchers to efficiently manage and access the extensive genomic information presented in the SesamumGDB.

### A case study of the application of SesamumGDB

**Figure 5.**
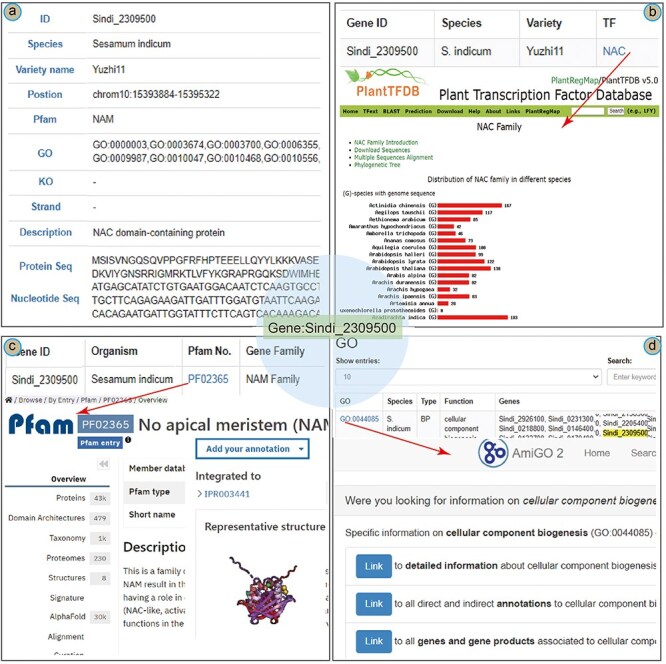
A case study of Sindi_2309500 gene in SesamumGDB, including gene profiles (a), transcription factor discovery (b), gene family exploration (c), and GO term search with database cross-referencing (d).

To illustrate the main functions of the database, we present a case study of the *NAC* (NAM/ATAF/CUC) gene in the SesamumGDB. The NAC gene *Sindi_2309500* was identified as crucial in modulating the upregulation of the oil content in sesame [3]. In the “Gene Retrieve” section, the profile of *Sindi_2309500* is organized into 11 categories (Fig. 5a). This gene is located on chromosome 10 of the sesame genome (var. Yuzhi 11), spanning from positions 15 393 884 bp to 15 395 322 bp and orientated on the negative strand. Pfam analysis has identified a “No apical meristem” (NAM) domain within this gene, and it is annotated as a NAC domain-containing protein [43, 44]. In addition, the profile includes detailed information regarding the gene’s nucleotide sequences and its corresponding amino acid composition.

As a TF, detailed information on *Sindi_2309500* is accessible through the “Transcription Factor” module (Fig. 5b). The exact match results include a hyperlink to the TF term “NAC,” providing an extensive overview of the NAC family based on PlantTFDB. These results elucidate the characteristics and distribution of the NAC encoded by *Sindi_2309500* across the *Sesamum* species. Furthermore, entering “NAC” into the search function yields a complete list of all 1001 NAC genes across the nine *Sesamum* genome.

Regarding *Sindi_2309500*, the gene search results highlighted the presence of a NAM domain (PF02365) in its protein sequence as a notable feature (Fig. 5a). This NAM domain could be further explored by navigating to the “Gene Families” module. Here, “NAM family” was entered as the search keyword, and “*Sesamum indicum*” was selected to specify the species. This targeted search approach yielded a succinct list of all 72 genes containing the NAM domain within the *S. indicum* genome. Additionally, clicking on the direct link to the InterPro database with the specific Pfam identifier [45], provided more detailed insights into the NAM domain’s functional and structural attributes (Fig. 5c).

In addition to basic search functionalities, the “GO” module in the SesamumGDB allows users to enter the specific gene identifiers to obtain detailed gene function information. For instance, upon entering the gene ID Sindi_2309500, we could retrieve a comprehensive list of associated GO terms, identifying a total of 41 distinct GO terms for *S. indicum* (var. Yuzhi11). By selecting an individual GO term, such as GO:0044085, users are directed to the AmiGO2 website where detailed information on the GO:0044085 term is presented (Fig. 5d).

The KEGG module of the SesamumGDB presents detailed pathway maps related to specific genes in a tabular format. For example, because *Sindi_2309500* lacks KEGG annotation, we randomly selected another gene, *Sindi_0007900*, for exploration within this module. When Sindi_0007900 was entered into the KEGG module, a set of five results were displayed in a tabular format. By clicking on a KO number in the table, such as ko00061, we obtained a detailed pathway map in KEGG database (Supplementary Fig. S1). In this case, the pathway map corresponded to the fatty acid biosynthesis pathway, illustrating the significance of SesamumGDB in the exploration of both function and related metabolic pathways associated with a gene. Furthermore, to ensure the effective utilization of SesamumGDB, we have provided a comprehensive tutorial accessible under the “Help” menu.

## Discussion and conclusion

Sesame, a crucial oilseed crop, occupies a unique position in the genome evolution of eudicot plants [3, 46]. In this study, we developed SesamumGDB, the first genome database specifically curated for the genus *Sesamum*. This database compiles genomic information for the single cultivated species and seven wild relatives. Moreover, this database lays the foundation for future pan-genome analyses and functional genomics and genetics research in sesame, especially considering the rich agronomic traits that have emerged through domestication and selective breeding processes [47]. Thus, the extensive genomic sources of *Sesamum* are expected to help resolve genetic bottlenecks associated with sesame domestication [48, 49].

As the first database for *Sesamum*, the SesamumGDB offers an extensive collection of genomic data from nine genomes across eight species, covering all three chromosome groups found in *Sesamum* (2*n* = 26, 32, and 64). Unlike existing sesame genome databases that primarily focus on the cultivated sesame species, the SesamumGDB provides a wealth of genomic information for the entire *Sesamum* genus. This includes comprehensive data on gene families, with a particular emphasis on crucial genes related to lipid biosynthesis and metabolism, as well as TFs (Fig. 1b).

Our commitment to updating the genome datasets has significantly facilitated gene discovery and genomic analysis for sesame. We anticipate an influx of more comprehensive sesame genome data in the near future. SesamumGDB, as an essential tool, is well-positioned to evolve and integrate these new genomic resources for sesame. We are dedicated to continuously updating the database and enhancing the robustness of genome information in the SesamumGDB, establishing it as a cornerstone for global sesame research.

## Supplementary Material

baae105_Supp

## References

[1] Miao H , Zhang H, and Kole C. *The Sesame Genome*. Cham, Switzerland: Springer, 2021.

[2] Bedigian D . *Sesame: The Genus Sesamum*. Boca Raton, FL, USA: CRC Press, 2010.

[3] Miao H , Wang L, Qu L et al. Genomic evolution and insights into agronomic trait innovations of *Sesamum* species. *Plant Commun* 2024;5:100729. doi: 10.1016/j.xplc.2023.10072 PMC10811377 37798879

[4] Yadav B , Thiruvengadam V, Sasikala R et al. Analysis of genetic diversity in sesame (*Sesamum indicum* L.) germplasm for yield and its attributing traits. *Electron J Plant Breed* 2022;13:927–31.

[5] Nimmakayala P Perumal R Mulpuri S et al. Sesamum. In: Kole C (ed.), *Wild Crop Relatives: Genomic and Breeding Resources: Oilseeds*. Berlin, Heidelberg: Springer, 2011, 261–73.

[6] Zhang H , Langham DR, and Miao H. Economic and academic importance of sesame. In: Miao H, Zhang H, Kole C (eds.), *The Sesame Genome, Compendium of Plant Genomes*. Cham, Switzerland: Springer 2021, 1–18. doi: 10.1007/978-3-319-98098-0_1

[7] Zhang H , Wang L, Miao H. Background of the sesame genome project. In: Miao H, Zhang H, Kole C (eds.), The Sesame Genome, Compendium of Plant Genomes. Cham, Switzerland: Springer, 2021, 199–204. doi: 10.1007/978-3-319-98098-0_10

[8] Zhang H , Miao H, Wang L et al. Genome sequencing of the important oilseed crop *Sesamum indicum* L. *Genome Biol* 2013;14:1–9. doi: 10.1186/gb-2013-14-1-401 PMC3663098 23369264

[9] Wang L , Yu S, Tong C et al. Genome sequencing of the high oil crop sesame provides insight into oil biosynthesis. *Genome Biol* 2014;15:1–13. doi: 10.1186/gb-2014-15-2-r39 PMC4053841 24576357

[10] Wang M , Huang J, Liu S et al. Improved assembly and annotation of the sesame genome. *DNA Res* 2022;29:dsac041. doi: 10.1093/dnares/dsac041 PMC9724774 36355766

[11] Song S , Dossou SSK, Meng M et al. Five improved sesame reference genomes and genome resequencing unveil the contribution of structural variants to genetic diversity and yield‐related traits variation. *Plant Biotechnol J* 2023;21:1722. 10.1111/pbi.14092 PMC10440982 37306179

[12] Wang X , Wang S, Lin Q et al. The wild allotetraploid sesame genome provides novel insights into evolution and lignan biosynthesis. *J Adv Res* 2023;50:13–24. 36265763 10.1016/j.jare.2022.10.004 PMC10403651

[13] Wang L , Yu J, Li D et al. Sinbase: an integrated database to study genomics, genetics and comparative genomics in *Sesamum indicum*. *Plant Cell Physiol* 2015;56:e2. 10.1093/pcp/pcu175 25480115

[14] Wei X , Gong H, Yu J et al. SesameFG: an integrated database for the functional genomics of sesame. *Sci Rep* 2017;7:2342. 10.1038/s41598-017-02586-3 PMC5443765 28539606

[15] Berhe M , Dossa K, You J et al. Genome-wide association study and its applications in the non-model crop *Sesamum indicum*. *BMC Plant Biol* 2021;21:283. 10.1186/s12870-021-03046-x PMC8218510 34157965

[16] Wei X , Liu K, Zhang Y et al. Genetic discovery for oil production and quality in sesame. *Nat Commun* 2015;6:8609. 10.1038/ncomms9609 PMC4634326 26477832

[17] Purru S , Sahu S, Rai S et al. GinMicrosatDb: a genome-wide microsatellite markers database for sesame (*Sesamum indicum* L.). *Physiol Mol Biol Plants* 2018;24:929–37. 30150867 10.1007/s12298-018-0558-8 PMC6103941

[18] Dossa K , Yu J, Liao B et al. Development of highly informative genome-wide single sequence repeat markers for breeding applications in sesame and construction of a web resource: SisatBase. *Front Plant Sci* 2017;8:1470. 10.3389/fpls.2017.01470 PMC5572293 28878802

[19] Benson G . Tandem repeats finder: a program to analyze DNA sequences. *Nucleic Acids Res* 1999;27:573–80. 9862982 10.1093/nar/27.2.573 PMC148217

[20] Xu Z , Wang H. LTR_FINDER: an efficient tool for the prediction of full-length LTR retrotransposons. *Nucleic Acids Res* 2007;35:W265–8. 17485477 10.1093/nar/gkm286 PMC1933203

[21] Ou S , Jiang N. LTR_retriever: a highly accurate and sensitive program for identification of long terminal repeat retrotransposons. *Plant Physiol* 2018;176:1410–22. 29233850 10.1104/pp.17.01310 PMC5813529

[22] Flynn JM , Hubley R, Goubert C et al. RepeatModeler2 for automated genomic discovery of transposable element families. *Proc Natl Acad Sci USA* 2020;117:9451–57. 32300014 10.1073/pnas.1921046117 PMC7196820

[23] Chen N . Using RepeatMasker to identify repetitive elements in genomic sequences. *Curr Protoc Bioinf* 2004;5:4.10.11–14. 10.1002/0471250953.bi0410s05 18428725

[24] Nishimura D . RepeatMasker. *Biotech Softw Internet Rep* 2000;1:36–39.

[25] Brůna T , Hoff KJ, Lomsadze A et al. BRAKER2: automatic eukaryotic genome annotation with GeneMark-EP+ and AUGUSTUS supported by a protein database. *NAR Genom Bioinform* 2021;3:lqaa108. 10.1093/nargab/lqaa108 PMC7787252 33575650

[26] Kim D , Paggi JM, Park C et al. Graph-based genome alignment and genotyping with HISAT2 and HISAT-genotype. *Nat Biotechnol* 2019;37:907–15. 31375807 10.1038/s41587-019-0201-4 PMC7605509

[27] Pertea M , Pertea GM, Antonescu CM et al. StringTie enables improved reconstruction of a transcriptome from RNA-seq reads. *Nat Biotechnol* 2015;33:290–95. 25690850 10.1038/nbt.3122 PMC4643835

[28] Haas BJ , Salzberg SL, Zhu W et al. Automated eukaryotic gene structure annotation using EVidenceModeler and the Program to Assemble Spliced Alignments. *Genome Biol* 2008;9:1–22. 10.1186/gb-2008-9-1-r7 PMC2395244 18190707

[29] Deng Y , Li J, Wu S et al. Integrated NR database in protein annotation system and its localization. *Comput Eng* 2006;32:71–72.

[30] Boeckmann B , Bairoch A, Apweiler R et al. The SWISS-PROT protein knowledgebase and its supplement TrEMBL in 2003. *Nucleic Acids Res* 2003;31:365–70. 12520024 10.1093/nar/gkg095 PMC165542

[31] Kanehisa M (ed). *The KEGG Database. ‘In Silico’ simulation of Biological Processes: Novartis Foundation Symposium*. Chichester, West Sussex, UK: Wiley Online Library, 2002, 247.

[32] Gene Ontology Consortium . The Gene Ontology (GO) database and informatics resource. *Nucleic Acids Res* 2004;32:D258–61. 14681407 10.1093/nar/gkh036 PMC308770

[33] Nawrocki EP , Eddy SR. Infernal 1.1: 100-fold faster RNA homology searches. *Bioinformatics* 2013;29:2933–35. 24008419 10.1093/bioinformatics/btt509 PMC3810854

[34] Chan PP , Lin BY, Mak AJ et al. tRNAscan-SE 2.0: improved detection and functional classification of transfer RNA genes. *Nucleic Acids Res* 2021;49:9077–96. 34417604 10.1093/nar/gkab688 PMC8450103

[35] Cantalapiedra CP , Hernández-Plaza A, Letunic I et al. eggNOG-mapper v2: functional annotation, orthology assignments, and domain prediction at the metagenomic scale. *Mol Biol Evol* 2021;38:5825–29. 34597405 10.1093/molbev/msab293 PMC8662613

[36] Mistry J , Bateman A, Finn RD. Predicting active site residue annotations in the Pfam database. *BMC Bioinformatics* 2007;8:1–14. 17688688 10.1186/1471-2105-8-298 PMC2025603

[37] Jin J , Tian F, Yang D-C et al. PlantTFDB 4.0: toward a central hub for transcription factors and regulatory interactions in plants. *Nucleic Acids Res* 2016:gkw982. 10.1093/nar/gkw982 PMC5210657 27924042

[38] Lohse M , Nagel A, Herter T et al. Mercator: a fast and simple web server for genome scale functional annotation of plant sequence data. Report No.: 0140-7791, Wiley Online Library, 2014. 10.1111/pce.12231 24237261

[39] Priyam A , Woodcroft BJ, Rai V et al. Sequenceserver: a modern graphical user interface for custom BLAST databases. *Mol Biol Evol* 2019;36:2922–24. 31411700 10.1093/molbev/msz185 PMC6878946

[40] Diesh C , Stevens GJ, Xie P et al. JBrowse 2: a modular genome browser with views of synteny and structural variation. *Genome Biol* 2023;24:74. 10.1186/s13059-023-02914-z PMC10108523 37069644

[41] Huerta-Cepas J , Szklarczyk D, Heller D et al. eggNOG 5.0: a hierarchical, functionally and phylogenetically annotated orthology resource based on 5090 organisms and 2502 viruses. *Nucleic Acids Res* 2019;47:D309–14. 30418610 10.1093/nar/gky1085 PMC6324079

[42] Gene Ontology Consortium . Gene ontology consortium: going forward. *Nucleic Acids Res* 2015;43:D1049–56. 25428369 10.1093/nar/gku1179 PMC4383973

[43] Puranik S , Sahu PP, Srivastava PS et al. NAC proteins: regulation and role in stress tolerance. *Trends Plant Sci* 2012;17:369–81. 22445067 10.1016/j.tplants.2012.02.004

[44] Wang Z , Dane F. NAC (NAM/ATAF/CUC) transcription factors in different stresses and their signaling pathway. *Acta Physiol Plant* 2013;35:1397–408.

[45] Paysan-Lafosse T , Blum M, Chuguransky S et al. InterPro in 2022. *Nucleic Acids Res* 2023;51:D418–27. 36350672 10.1093/nar/gkac993 PMC9825450

[46] Weldemichael MY , Gebremedhn HM. Omics technologies towards sesame improvement: a review. *Mol Biol Rep* 2023;50:6885–99. 37326753 10.1007/s11033-023-08551-w

[47] Tripathy SK Kar J , and Sahu D. Advances in sesame (*Sesamum indicum* L.) breeding. In: Al-Khayri JM, Jain SM, Johnson DV (eds.), *Advances in Plant Breeding Strategies: Industrial and Food Crops*, Vol. 6. Cham, Switzerland: Springer, 2019, 577–635.

[48] Teklu DH , Shimelis H, Abady S. Genetic improvement in sesame (*Sesamum indicum* L.): progress and outlook: a review. *Agronomy* 2022;12:2144.

[49] Pathak N , Rai AK, Saha S et al. Quantitative dissection of antioxidative bioactive components in cultivated and wild sesame germplasm reveals potentially exploitable wide genetic variability. *J Crop Sci Biotechnol* 2014;17:127–39.

