# Supplementary figures and images for: SesamumGDB: a comprehensive platform for Sesamum genetics and genomics analysis
Source: Database (Oxford). 2024 Oct 19;2024:baae105. doi: 10.1093/database/baae105 (PMC11490215; doi:10.1093/database/baae105)

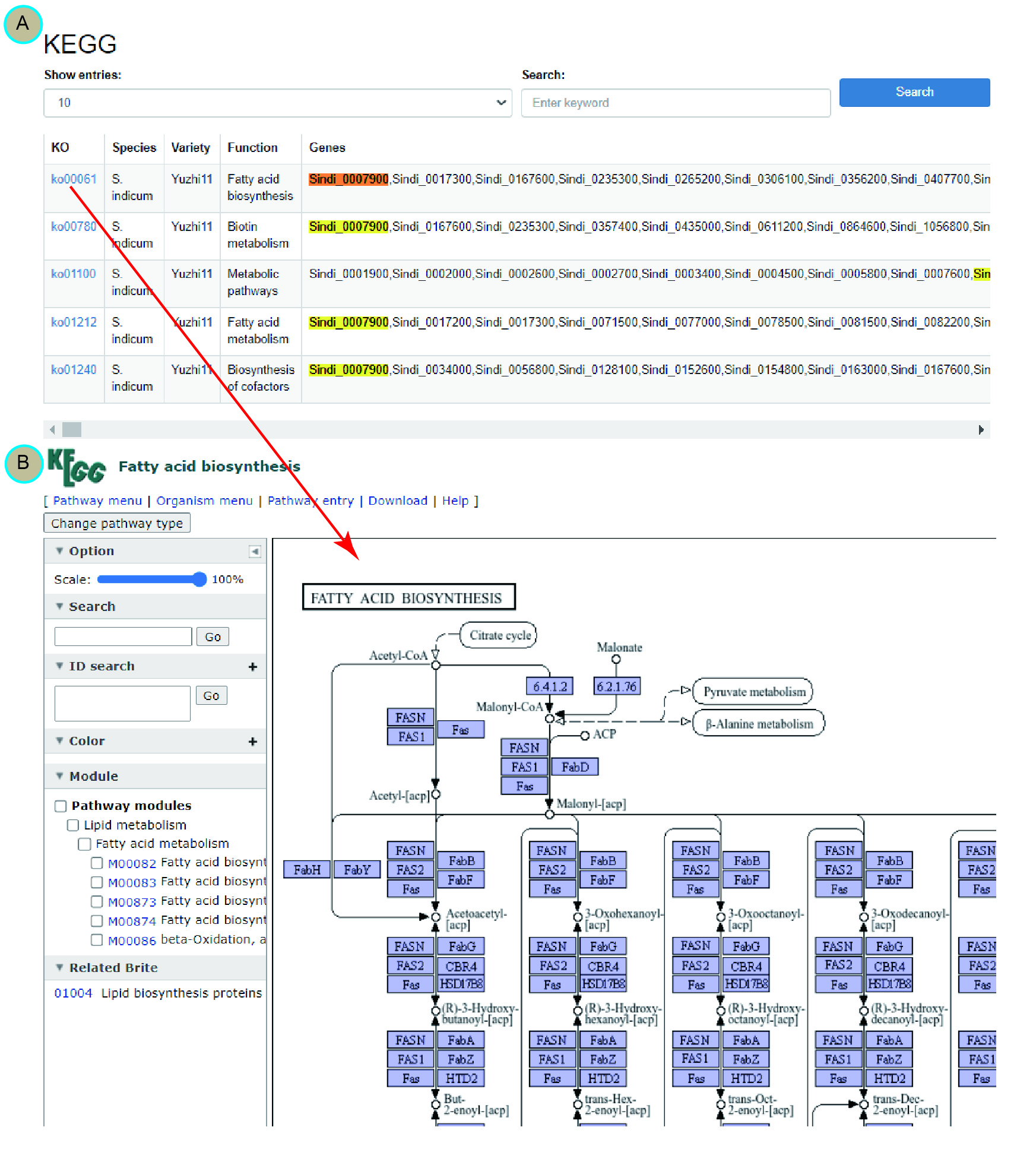

Supplement: baae105_Supp [file baae105_supp.zip › suppl_data/Fig S1 .jpg]
